# Harnessing the genomic diversity of *Pseudomonas* strains against lettuce bacterial pathogens

**DOI:** 10.3389/fmicb.2022.1038888

**Published:** 2022-12-22

**Authors:** Antoine Zboralski, Adrien Biessy, Marie Ciotola, Mélanie Cadieux, Daphné Albert, Jochen Blom, Martin Filion

**Affiliations:** ^1^Centre de Recherche et de Développement de Saint-Jean-sur-Richelieu, Agriculture et Agroalimentaire Canada, Saint-Jean-sur-Richelieu, QC, Canada; ^2^Bioinformatics and Systems Biology, Justus Liebig University Giessen, Giessen, Germany

**Keywords:** *Pseudomonas*, *Xanthomonas*, *Pectobacterium*, lettuce, PGPR, pathogen, genome, biocontrol

## Abstract

Lettuce is a major vegetable crop worldwide that is affected by numerous bacterial pathogens, including *Xanthomonas hortorum* pv. *vitians*, *Pseudomonas cichorii*, and *Pectobacterium carotovorum*. Control methods are scarce and not always effective. To develop new and sustainable approaches to contain these pathogens, we screened more than 1,200 plant-associated *Pseudomonas* strains retrieved from agricultural soils for their *in vitro* antagonistic capabilities against the three bacterial pathogens under study. Thirty-five *Pseudomonas* strains significantly inhibited some or all three pathogens. Their genomes were fully sequenced and annotated. These strains belong to the *P*. *fluorescens* and *P*. *putida* phylogenomic groups and are distributed in at least 27 species, including 15 validly described species. They harbor numerous genes and clusters of genes known to be involved in plant-bacteria interactions, microbial competition, and biocontrol. Strains in the *P*. *putida* group displayed on average better inhibition abilities than strains in the *P*. *fluorescens* group. They carry genes and biosynthetic clusters mostly absent in the latter strains that are involved in the production of secondary metabolites such as 7-hydroxytropolone, putisolvins, pyochelin, and xantholysin-like and pseudomonine-like compounds. The presence of genes involved in the biosynthesis of type VI secretion systems, tailocins, and hydrogen cyanide also positively correlated with the strains’ overall inhibition abilities observed against the three pathogens. These results show promise for the development of biocontrol products against lettuce bacterial pathogens, provide insights on some of the potential biocontrol mechanisms involved, and contribute to public *Pseudomonas* genome databases, including quality genome sequences on some poorly represented species.

## 1 Introduction

Lettuce (*Lactuca sativa*, *Asteraceae*) is a widely consumed vegetable worldwide that generated in 2018 a world gross production value of 14.3 billion USD, when combined with chicory (Food and Agriculture Organization of the United Nations, 2021). However, production is affected by several bacterial and fungal pathogens in most growing regions of the world, often resulting in important yield and economic losses (Subbarao et al., 2017). Some of the main bacterial pathogens of lettuce include *Xanthomonas hortorum* pv. *vitians* (Hébert et al., 2021), formerly known as *X. campestris* pv. *vitians* (Vauterin et al., 1995; Morinière et al., 2020), *Pseudomonas cichorii* (Pauwelyn et al., 2011), and *Pectobacterium carotovorum* (Cariddi and Sanzani, 2013). These three bacteria directly affect lettuce aboveground tissues, especially leaves.

*X*. *hortorum* pv *vitians* is a hemibiotrophic organism mainly infecting plant species belonging to the *Lactuca* genus, especially lettuce, on which it induces bacterial leaf spot (Toussaint et al., 2012; Morinière et al., 2022). This disease is characterized by the development of water-soaked and geometric lesions on leaves (Subbarao et al., 2017; Hébert et al., 2021). These lesions can expand and coalesce, leading to the necrosis of entire leaves. Worldwide, this pathogen has been responsible for several outbreaks on lettuce over the past decade and is considered re-emergent (Subbarao et al., 2017; Morinière et al., 2022). Infection is T3SS-dependent and relies on the secretion of cell wall degrading-enzymes, the use of type VI secretion systems (T6SS) and their effectors, as well as other determinants (Büttner and Bonas, 2010; Timilsina et al., 2020). *X*. *hortorum* pv *vitians* penetrates the leaves through natural openings, mainly the stomata and the hydrathodes, and through wounds. The pathogen then colonizes the mesophyll, before reaching the vascular system (Dia et al., 2022).

*P*. *cichorii*, another hemibiotrophic pathogen, infects various hosts and causes varnish spot on lettuce (also called midrib rot) (Hikichi et al., 2013). This disease consists in the development of firm dark-brown necrotic lesions on inner leaves of head-forming lettuce cultivars, sometimes along veins and midribs (Subbarao et al., 2017). *P*. *cichorii* produces numerous phytotoxic compounds to infect the plant, such as lipopeptides, and sometimes uses a type III secretion system (T3SS) as well, leading to plant cell death (Hikichi et al., 2013; Huang et al., 2015). It enters the leaves through the stomata, develops into intercellular spaces of the leaf epidermis and the mesophyll, until reaching the vascular system, in a similar way to *X*. *hortorum* pv *vitians* (Hikichi et al., 2013).

*P. carotovorum* is a necrotrophic pathogen able to infect a broad range of plant species, including lettuce, on which it causes bacterial soft rot (Cariddi and Sanzani, 2013; Davidsson et al., 2013). This disease is characterized by the wilting of the outer leaves and a macerated gelatinous greenish pith, eventually leading to the rotting of the entire lettuce plant (Subbarao et al., 2017). Pathogenicity determinants of *P*. *carotovorum* include a wide array of plant cell wall-degrading enzymes, along with some toxins (Davidsson et al., 2013). The pathogen infects its hosts primarily through wounds (Agrios, 2005).

The current strategies to control these pathogens are often ineffective and rely primarily on preventive methods, as few curative options are available, if any (Pauwelyn et al., 2011; Cariddi and Sanzani, 2013; Subbarao et al., 2017; Hébert et al., 2021). Prophylactic methods include the use of crop rotation, less susceptible cultivars, seed and equipment cleaning, well-drained soils, adequate spacing between the plants to improve air movement and reduce leaf wetness, and drip irrigation instead of sprinkler irrigation (Agrios, 2005; Subbarao et al., 2017). Copper-based compounds have been used to try to control lettuce bacterial diseases such as bacterial leaf spot (Dia et al., 2022). However, these compounds sometimes cause phytotoxicity and their widespread use favors resistance emergence, leading to restricted usage (Carisse et al., 2000; Fatmi and Bolkan, 2017; Dia et al., 2022). A fungicide containing potassium salts of phosphorous acid is also used to control bacterial pathogens on lettuce (Hébert et al., 2021). It favors resistance development in bacterial populations as well. To decrease the impacts of bacterial pathogens on lettuce, more efficient, sustainable, and environmentally friendly methods need to be developed and implemented.

Biocontrol could become the cornerstone of these new strategies. It consists in using living agents to control detrimental organisms in order to provide human benefits (Stenberg, 2021). In plant pathology, it especially pertains to the direct or indirect inhibition of a pathogen, or the symptoms it causes, by another organism or a consortia of organisms (Cook and Baker, 1983). Such inhibition can be mediated by antibiosis, hyperparasitism, the induction of plant defenses, and competition for resources (Collinge et al., 2022). Biocontrol offers several advantages compared to compound-based pesticides, such as reduced risks of soil contamination by harmful residues, various modes of action limiting the emergence of resistance in pathogens, and a potentially better social acceptability (Collinge et al., 2022). Some bacteria living in the rhizosphere of plants have been described as effective biocontrol agents. They are often called plant growth-promoting rhizobacteria (PGPR), alongside rhizobacteria that directly improve plant growth by enhancing plant nutrition or interfering with plant hormone signaling (Lugtenberg and Kamilova, 2009).

*Pseudomonas* is amongst the most studied PGPR-containing genera (Weller, 2007; Höfte, 2021). It belongs to the recently renamed *Pseudomonadota* phylum, previously called *Proteobacteria* (Oren and Garrity, 2021). The *Pseudomonas* genus includes bacteria that are rod-shaped, motile, Gram-negative, mostly aerobic, and distributed across many habitats and hosts (Palleroni, 1984). Many *Pseudomonas* strains displaying biocontrol abilities against a myriad of bacterial, fungal, and oomycete plant pathogens have been identified over the past decades, especially from agricultural soils (Weller, 2007; Biessy et al., 2019, 2021; Balthazar et al., 2021). A multitude of them belong to the *P*. *fluorescens* phylogenomic group, and to a lesser extent, to the *P*. *putida* group (Weller, 2007; Berendsen et al., 2015; Planchamp et al., 2015; Biessy et al., 2019; Höfte, 2021). They are able to produce diverse biocontrol-related secondary metabolites, such as antibiotics, lipopeptides, and siderophores, as well as molecules involved in plant-bacteria interactions, including hormones and effectors (Gross and Loper, 2009; Ghequire and De Mot, 2014; Götze and Stallforth, 2020; Zboralski and Filion, 2020; Zboralski et al., 2022). Only few *Pseudomonas* strains have been identified as effective biocontrol agents against lettuce pathogens. To our knowledge, these have mostly been assessed against fungal pathogens, not bacterial ones (Grosch et al., 2005; Sottero et al., 2006; Adesina et al., 2007; Scherwinski et al., 2008; Schreiter et al., 2018; Aggeli et al., 2020).

To address this issue and to contribute to the development of sustainable biocontrol solutions against lettuce bacterial pathogens, we collected more than 1,200 *Pseudomonas* strains from agricultural soils in the province of Québec (Canada). We identified 35 strains displaying promising biocontrol abilities against *X*. *hortorum* pv. *vitians*, *P*. *cichorii*, and/or *P*. *carotovorum*. The genomes of these strains were sequenced and analyzed to identify biosynthetic genes known to be involved in the production of secondary metabolites of interest in biocontrol.

## 2 Materials and methods

### 2.1 Bacterial strains isolation

All *Pseudomonas* strains used in this study were isolated in 2019 from agricultural soils collected from 29 polyculture organic farms located in the Montérégie region in Québec, Canada. Soil samples were collected close to the roots of plants belonging to various vegetable species and stored at 4^°^C. One gram of each soil sample was diluted into 100 ml of a 0.9% NaCl solution and the resulting suspension was shaken for 10 min at 250 rpm. Serial dilutions of this suspension were spread on King’s B agar medium (King et al., 1954) amended with 100 μg.ml^–1^ cycloheximide, 40 μg.ml^–1^ ampicillin, and 13 μg.ml^–1^ chloramphenicol to select for *Pseudomonas* strains (McSpadden Gardener et al., 2001). Plates were incubated for 48 h at 25^°^C. Single colonies were retrieved and successively streaked several times on regular King’s B agar medium to ensure purity. The strains were cryopreserved at –80^°^C in tryptic soy broth with 10% glycerol until further use.

### 2.2 *In vitro* confrontational assays against bacterial lettuce pathogens

The inhibition capabilities of all isolates were individually assessed *in vitro* against each lettuce bacterial pathogen, namely *X*. *hortorum* pv. *vitians* B07-007, *P*. *cichorii* B12-019, and *P*. *carotovorum* B12-025. These strains were isolated from infected lettuce leaves in Southern Quebec, Canada (Affia, 2016; Hébert et al., 2021). Each pathogenic bacterium was initially grown for 48 h at 22.5^°^C on yeast dextrose carbonate agar medium for *X*. *hortorum* pv. *vitians* and on King’s B agar medium for the other two pathogens. The bacteria were then resuspended from the plate into sterile distilled water, to a final concentration of 10^8^ CFU.ml^–1^. These suspensions of bacterial pathogens were used in the following assays.

A screening of all *Pseudomonas* isolates was performed using a modified perpendicular streaking method (Gislin et al., 2018). *Pseudomonas* strains were grown in King’s B broth for 24 h at 125 rpm and 22.5^°^C before being directly streaked using an inoculation loop at the bottom of Petri plates containing tryptic soy agar (TSA) medium. After 48 h, a suspension volume of 10 μl of each of the three bacterial pathogens adjusted to 10^8^ CFU.ml^–1^, prepared as described above, was applied on the top of each Petri plate. The Petri dish was immediately inclined to allow the three suspensions of pathogens to reach the streaked *Pseudomonas* strain. Plates were incubated for 5 days at 25^°^C and were subsequently evaluated for any antagonistic effect.

*Pseudomonas* isolates that were found to have an antagonistic activity against at least one pathogen were further used. Their antagonistic effect was quantitatively assessed using an overlay method. This method consisted in applying the antagonistic *Pseudomonas* strains directly onto the pathogenic bacteria already spread all over the plate. Briefly, a volume of 100 μl of each bacterial pathogen suspension was individually spread onto TSA medium to fully cover the Petri’s surface and left to dry for 2 h. Each antagonistic *Pseudomonas* strain was grown for 24 h on King’s B agar medium and resuspended in distilled water to a final concentration of 10^8^ CFU.ml^–1^. Twenty microliters were then added to the center of the plate. After 6 days at 25^°^C, the diameter of the entire inhibition zone was measured, namely the circular area where the pathogen was not able to grow. Three plates were prepared for each pathogen-*Pseudomonas* isolate combination.

### 2.3 Genome sequencing, assembly, and annotation

Isolates displaying any inhibitory activity against at least one of the three bacterial pathogens were selected for whole-genome sequencing. Their DNA was extracted from bacterial cells grown in King’s B agar medium for 48 h at 25^°^C using the DNeasy UltraClean microbial kit (Qiagen, Toronto, ON, Canada) following a modified version of the manufacturer’s instructions. An additional step was performed just before mechanical lysis of the samples to improve the yield: 10 μl of proteinase K (Bioshop Canada, Burlington, ON, Canada) were added to each sample and the samples were then heated 10 min at 70^°^C before proceeding with the manufacturer’s instructions. The preparation of the genomic DNA libraries as well as the sequencing were carried out at the Integrated Microbiome Resource (Halifax, NS, Canada). Genomic DNA was mechanically sheared to obtain 9–10 kb fragments using Covaris g-TUBE (Covaris, CA). Libraries were prepared using the PacBio SMRTbell Express Template Prep kit (Pacific Biosciences, Menlo Park, CA). The sequencing was accomplished with a PacBio Sequel sequencer (v3 chemistry). The genomes were assembled using the long-read assembler Flye v2.8.1 (Kolmogorov et al., 2019). Circular contigs were rotated using Geneious Prime 2022.1.1 (Biomatters, Auckland, New Zealand). Annotation was carried out by the NCBI Prokaryotic Genome Annotation Pipeline v5.3 (Tatusova et al., 2016).

### 2.4 Species-level identification

Species-level identification of the strains under study was performed using the Type (Strain) Genome Server (Meier-Kolthoff and Göker, 2019; Meier-Kolthoff et al., 2022). This web server provides digital DNA-DNA hybridization (dDDH) values between a query genome and a set of closely related type strain genomes. dDDH values were calculated using the Genome BLAST Distance Phylogeny (GBDP) formula d_4_ (Meier-Kolthoff et al., 2013). When the dDDH value between a strain and a type strain was slightly below the 70% threshold, the JSpeciesWS online tool (Richter et al., 2016) was used to calculate additional overall genome relatedness indexes (OGRIs), such as ANIb and TETRA.

### 2.5 Phylogenomic analyses

The web-server EDGAR 3.0 was used for ortholog calculation and phylogenomic analyses (Blom et al., 2009; Dieckmann et al., 2021). Orthologs were identified using BLAST bidirectional best hits with an orthology criterion corresponding to a score ratio value of 0.3. A phylogenomic tree was built out of 2,215 core genes per genome (79,740 in total) for a total of 2,163,293 bp per genome. The coding nucleotide sequences of the orthologous gene sets found in all genomes were individually aligned using MUSCLE (Edgar, 2004). The resulting alignments were concatenated into one that was used to generate the phylogenomic tree with the neighbor-joining method implemented in PHYLIP (Felsenstein, 2005). To verify the resulting tree topology, a bootstrapping was performed with 500 iterations. All branches within the tree showed 100% bootstrap support.

### 2.6 Identification of genes and BGCs involved in the production of phytobeneficial secondary metabolites

Genes and biosynthetic gene clusters involved in the production of secondary metabolites related to biocontrol, plant-growth promotion and rhizocompetence were searched for in all 35 genomes. Sequences of genes and biosynthetic gene clusters (BGCs) of interest were retrieved from GenBank and from the Pseudomonas genome database (Winsor et al., 2016). These sequences (Supplementary Table 1) were used as baits in BLAST searches to identify numerous genes and/or BGCs in the genomes of the strains under study. Putative homologs were identified using a cutoff of 70% sequence identity over 70% of the sequence length. Additional secondary metabolite BGCs were also identified using antiSMASH v6.0 (Blin et al., 2021). Antibacterial proteins (except for type VI effectors) were identified by a proteome-wide analysis of Pfam domains. This analysis was conducted using the built-in Pfam domain search v1.2 in CLC Genomics Workbench v21.0.5 (Qiagen, Aarhus, Denmark) and the Pfam-A v35.0 database (Mistry et al., 2021). Proteins harboring Pfam domains of interest were subsequently retrieved and subjected to further investigation with InterProScan v2.0 (Quevillon et al., 2005) in Geneious Prime 2022.1.1 (Biomatters, Auckland, New Zealand). Putative type VI effectors were identified in a two-step process. Firstly, genes encoding T6SS components, such as VgrG (PF04717/PF05954), Hcp (PF05638), PAAR domain-containing (PF05488) and TssB (PF05591) proteins, as well as type VI adaptor proteins (PF08786, PF09937, and PF13503), were mapped on the genomes of the strains under study. Then, potential type VI effectors encoded in the vicinity of these type VI-related genes were retrieved, analyzed with InterProScan, and compared to a collection of already characterized type VI effectors.

### 2.7 Statistical analyses

Data from confrontational assays was analyzed using R v3.6.1 with RStudio v1.2.5001 (R Studio Team, 2019; R Core Team, 2021). Multiple comparisons were performed through the “kruskal” function from the “agricolae” R package v1.3.1, involving Fisher’s least significant difference procedure with a Benjamini-Hochberg correction to adjust the *p*-values (de Mendiburu, 2017). Associations between genomic data and inhibition levels were determined using the Wilcoxon-Mann-Whitney test by comparing two groups, each containing at least 5 strains (Zboralski et al., 2020; Biessy et al., 2021). The level of significance was set at 0.05 for *p*-values, unless stated otherwise.

## 3 Results

### 3.1 Thirty-five *Pseudomonas* strains displaying biocontrol potential were isolated

**FIGURE 1:**
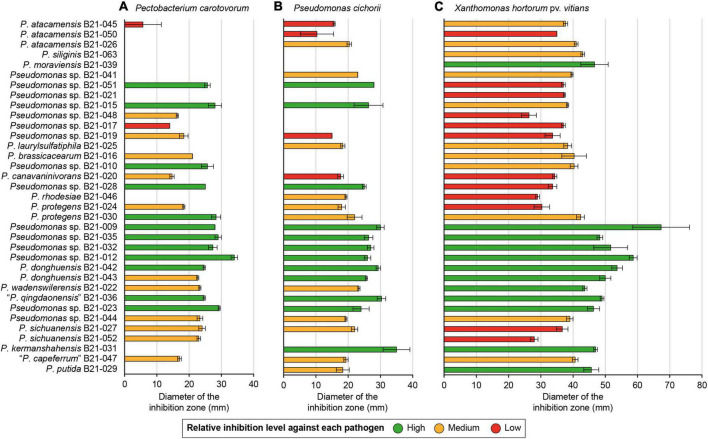
*In vitro* inhibition abilities of 35 *Pseudomonas* strains inhibiting *P*. *carotovorum* **(A)**, *P*. *cichorii* **(B)**, and/or *X*. *hortorum* pv. *vitians* **(C)**. The colored inhibition levels against a given pathogen are significantly distinct from each other regarding the “High” and “Low” group (*p* < 0.001, Fisher’s least significant difference procedure). The “Medium” group strains do not necessarily display any significant difference in terms of inhibitory activity with strains from the other two groups. Strains are sorted according to their phylogenetic relationships. Error bars: standard error.

A total of 1,210 *Pseudomonas* strains were retrieved from agricultural soil samples and screened for *in vitro* antagonistic abilities against *X*. *hortorum* pv. *vitians*, *P*. *cichorii*, and *P*. *carotovorum*. The streaking technique was found to be an effective screening method that allowed to single out 35 *Pseudomonas* strains able to inhibit at least one of the lettuce bacterial pathogens under study (data not shown). The overlay method provided quantitative inhibition levels for these strains against the selected lettuce pathogenic strains (Figure 1). All strains were able to inhibit *X*. *hortorum* pv. *vitians* to some extent, while some strains displayed no inhibition against *P. cichorii* or *P*. *carotovorum*. Strains with low- and high-inhibition abilities were divided into two statistically distinct groups, and a third group included strains with intermediate inhibition abilities, which were not necessarily different from the abilities of the other two groups (Figure 1). Some strains belonged to the high-inhibition group for all three pathogens: B21-009, B21-012, B21-023, B21-032, B21-035, B21-036, and B21-042. Similarly, others belonged to the low-inhibition group for all three pathogens: B21-017, B21-021, and B21-050. Some strains were completely unable to generate an inhibition zone against *P*. *carotovorum* and *P*. *cichorii*, while generating one against *X*. *hortorum* pv. *vitians*: B21-021, B21-039, and B21-063.

### 3.2 Sequencing reveals diverse *Pseudomonas* species among the inhibitory strains

The genomes of the 35 strains were sequenced on a PacBio Sequel sequencer, yielding an average of 286,069 reads with an average length of 5,333 bp (Supplementary Table 2). The long-read assembler Flye managed to completely assemble each of the 35 genomes into a single circular chromosome. Genome size ranges from 5.58 to 7.07 Mb (average 6.14 Mb) and the GC content varies from 58.5 to 64.3% (average 61.5%). Details on all sequenced genomes are provided in Supplementary Tables 2, 3. The GenBank accession numbers are displayed in Table 1.

**TABLE 1:** List of the 35 sequenced *Pseudomonas* strains used in this study, according to their phylogenomic groups and subgroups.

Phylogenomic group	Phylogenomic subgroup	Species name	Strain	GenBank accession number
*P*. *fluorescens*	*P*. *koreensis*	*P*. *atacamensis*	B21-045	CP087171
		*P*. *atacamensis*	B21-050	CP087167
		*P*. *atacamensis*	B21-026	CP087187
		*P*. *siliginis*	B21-063	CP087186
		*P*. *moraviensis*	B21-039	CP087177
		*Pseudomonas* sp.	B21-041	CP087175
		*Pseudomonas* sp.	B21-051	CP087166
		*Pseudomonas* sp.	B21-021	CP087192
	*P*. *mandelii*	*Pseudomonas* sp.	B21-015	CP087196
		*Pseudomonas* sp.	B21-048	CP087168
		*Pseudomonas* sp.	B21-017	CP087194
		*Pseudomonas* sp.	B21-019	CP087193
	*P*. *jessenii*	*P*. *laurylsulfatiphila*	B21-025	CP087188
	*P*. *corrugata*	*P*. *brassicacearum*	B21-016	CP087195
		*Pseudomonas* sp.	B21-010	CP087198
		*P*. *canavaninivorans*	B21-020	CP102179
		*Pseudomonas* sp.	B21-028	CP087184
	*P*. *fluorescens*	*P*. *rhodesiae*	B21-046	CP087170
	*P*. *protegens*	*P*. *protegens*	B21-024	CP087189
		*P*. *protegens*	B21-030	CP087182
*P*. *putida*		*Pseudomonas* sp.	B21-009	CP087199
		*Pseudomonas* sp.	B21-035	CP087179
		*Pseudomonas* sp.	B21-032	CP087180
		*Pseudomonas* sp.	B21-012	CP087197
		*P*. *donghuensis*	B21-042	CP087174
		*P*. *donghuensis*	B21-043	CP087173
		*P*. *wadenswilerensis*	B21-022	CP087191
		*“P*. *qingdaonensis”*	B21-036	CP087178
		*Pseudomonas* sp.	B21-023	CP087190
		*Pseudomonas* sp.	B21-044	CP087172
		*P*. *sichuanensis*	B21-027	CP087185
		*P*. *sichuanensis*	B21-052	CP087165
		*P. kermanshahensis*	B21-031	CP087181
		*“P. capeferrum”*	B21-047	CP087169
		*P*. *putida*	B21-029	CP087183

The data used to identify these strains at the species level are provided in Supplementary Table 2.

**FIGURE 2:**
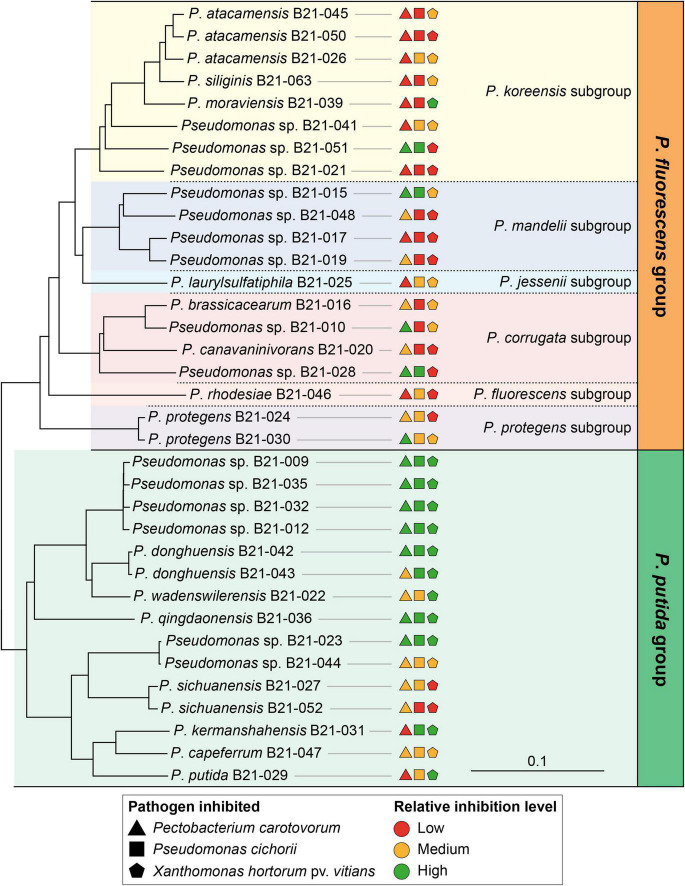
Neighbor-joining phylogeny of the 35 isolated *Pseudomonas* strains and their relative inhibition levels against various lettuce bacterial pathogens. This phylogenomic tree was generated with EDGAR from a concatenated alignment of 2,215 shared genes per genome. *P*. *aeruginosa* DSM 50071^T^ was used as an outgroup (not shown on the tree). The colored inhibition levels refer to the levels presented in Figure 1. All branches in the tree show 100% bootstrap support (500 iterations).

The 35 strains fall into two distinct phylogenomic groups within the genus *Pseudomonas*: the *P*. *fluorescens* (20 strains) and *P*. *putida* (15 strains) groups (Supplementary Figure 1). Within the *P*. *fluorescens* group, the 20 strains are distributed in six distinct subgroups: the *P*. *koreensis*, *P*. *mandelii*, *P*. *jessenii*, *P*. *corrugata*, *P*. *fluorescens*, and *P*. *protegens* subgroups (Figure 2 and Supplementary Figure 1). The Type (Strain) Genome Server enabled us to assign 20 strains to 15 distinct species (Supplementary Figure 1 and Supplementary Table 2). The 15 remaining strains do not belong to any species described to date for which a genome is publicly available. They potentially represent 12 new species (Supplementary Figures 1, 2).

### 3.3 The genomes contain genes and clusters involved in biocontrol and other plant growth-promoting traits

**FIGURE 3:**
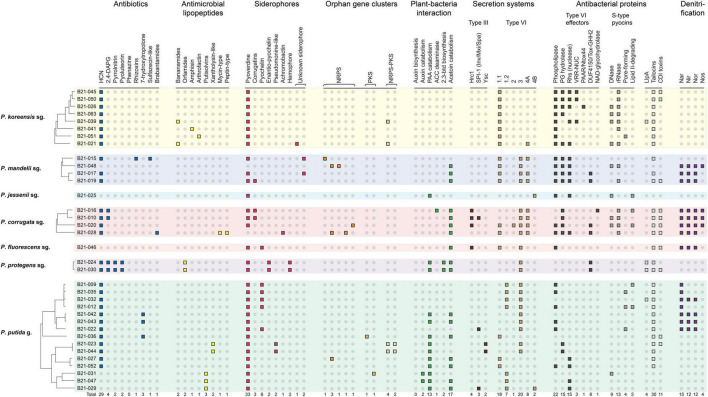
Distribution of diverse genes and clusters involved in plant-bacteria interactions, microbial competition, and biocontrol in the genomes of the 35 *Pseudomonas* strains. HCN, hydrogen cyanide; 2,4-DAPG, 2,4-diacetylphloroglucinol; NRPS, non-ribosomal peptide synthase; PKS, polyketide synthase; PAA, phenylacetic acid; ACC, 1-aminocyclopropane-1-carboxylate; 2,3-btd, 2,3-butanediol; PG, peptidoglycan; Rhs, VRR-NUC, PAAR, Ntox44, DUF4150, and Tox-GHH2, Pfam domains harbored by some of the Type VI effectors; NAD, Nicotinamide adenine dinucleotide; Llpa, lectin-like putidacin A; CDI, contact-dependent inhibition; sg., subgroup; g., group.

The 35 strains under study display an important genomic diversity, as evidenced by the size of the pangenome, which reaches 19,863 protein-coding genes. The strains share only 2,364 orthologous coding sequences (CDSs), which account for about 37–47% of the total number of CDSs found in each genome (Supplementary Table 3). In addition, each strain harbors, on average, 124 singletons (Supplementary Table 3). Genes and BGCs involved in the production of secondary metabolites related to biocontrol, plant-growth promotion and rhizocompetence were searched in the 35 genomes under study to determine if this genomic diversity includes a diversity of biocontrol-associated traits. Various genes and/or gene clusters of interest, known to be involved in the biosynthesis of antibiotics, antimicrobial lipopeptides and proteins, siderophores, secretion system apparatuses, and plant hormone-related molecules were uncovered (Figure 3 and Supplementary Tables 4, 5).

Twenty-nine out of the 35 strains carry BGCs involved in the production of one to four antibiotics. These strains all potentially produce hydrogen cyanide, and some of them carry the gene clusters involved in 2,4-diacetylphloroglucinol, pyrrolnitrin, pyoluteorin, rhizoxins, 7-hydroxytropolone, and/or brabantamides synthesis.

Genes involved in siderophore production were found in all strains except B21-048, which even lacks the biosynthetic genes to produce pyoverdine. Sixteen strains also carry BGCs responsible for the biosynthesis of one or two additional siderophores. Six strains, mainly belonging to the *P*. *putida* group, carry the BGC involved in pyochelin production. BGCs putatively responsible for the synthesis of two unknown siderophores were also detected in three strains.

BGCs involved in the production of antimicrobial lipopeptides were found in 12 out of 35 strains. They are predicted to encode lipopeptides with various structures, containing between 8 and 22 amino acids.

As for secretion systems and antibacterial proteins, T3SS clusters were detected in eight strains and belong to three distinct families, i.e., Hrc1, SPI-1 (Inv/Mxi/Spa), and Ysc (Supplementary Figure 3). One strain, B21-010, carries two distinct T3SS clusters. Fifty-six T6SS clusters were found in the genomes of the 35 strains under study. Each strain harbors at least one T6SS cluster and up to four distinct ones. These T6SS clusters belong to six different groups (Supplementary Figure 4). Diverse antibacterial proteins were uncovered in the genomes, including type VI effectors and S-type pyocins, as well as contact-dependent inhibition systems and tailocins (Supplementary Table 5). Some strains harbor up to four S-type pyocins, and up to nine type VI effectors. Three strains carry neither S-type pyocin nor type VI effector genes: B21-032, B21-042, and B21-036. In addition, we found genes potentially involved in the biosynthesis of tailocins in the *mutS*–*cinA* intergenic region in 31 strains.

### 3.4 Phylogenetic relationships and some genomic traits correlate with inhibition abilities

**FIGURE 4:**
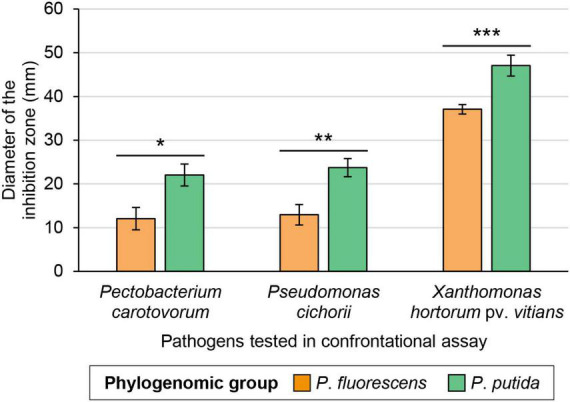
Average *in vitro* inhibition abilities of the *Pseudomonas* strains against three lettuce bacterial pathogens according to their phylogenomic group—either *P*. *fluorescens* (*n* = 20) or *P*. *putida* (*n* = 15). One, two, or three asterisks refer to *p*-values below 0.05, 0.01, and 0.001, respectively (Wilcoxon-Mann-Whitney test). Error bars, standard error.

Strains belonging to the *P*. *putida* phylogenomic group displayed significantly larger inhibition zones on average than those from the *P*. *fluorescens* group, against all bacterial pathogens under study (Figures 2, 4). Strains from the *P*. *putida* group generated inhibition zones on average 27% larger than those from the *P*. *fluorescens* group when tested against *X*. *hortorum* pv. *vitians*, and 83% larger against *P*. *cichorii* and *P*. *carotovorum*.

To better appreciate the potential contribution of some traits in pathogen inhibition abilities, we compared inhibition abilities of strains carrying a given gene or BGC (Figure 3) against strains not carrying it. The results are presented in Table 2. Five BGCs were positively associated with the ability to generate an inhibition zone against one or more of the tested pathogens. The genes involved in the biosynthesis of hydrogen cyanide (HCN), tailocins, and a group-3-type T6SS positively correlated with the ability of inhibiting *P*. *carotovorum*, and no other pathogen. The strains carrying the *hcn* cluster, encoding enzymes required for HCN biosynthesis, produced an inhibition zone 3.3 times larger than the strains not carrying it. The BGC of the group-2 T6SS was linked to higher inhibition levels against both *P*. *cichorii* and *X*. *hortorum* pv. *vitians*. The pyochelin BGC was positively associated with inhibition abilities against all three pathogens.

**TABLE 2:** Mean inhibition ratios and associated *p*-values between strains displaying and strains not displaying a given gene or gene cluster in their genomes, considering inhibition against *P*. *carotovorum*, *P*. *cichorii*, or *X*. *hortorum* pv. *vitians*.

Category	Gene or cluster	Mean inhibition ratio		
		***P*. *carotovorum***	***P*. *cichorii***	***X*. *hortorum* pv. *vitians***
Antibiotics	HCN	3.34*	–	–
Siderophore	Pyochelin	1.59*	1.59*	1.26*
Plant-bacteria interaction	Acetoin catabolism	–	–	0,87*
Type VI secretion system	1.1	0.59*	–	0.80***
	1.2	–	1.68*	1.32**
	3	2.82***	–	–
	4A	–	0.53*	0.86*
Antibacterial protein	PG hydrolase	–	0.52**	0.85*
	Rhs (nuclease)	0.57*	0.60**	0.83**
	DUF4150/Tox-GHH2	–	–	0.83*
	Dnase	0.32**	0.5*	–
	rRNase	0.48*	0.44***	–
	Tailocins	2.69*	–	–

Only genes or clusters of genes displaying a *p*-value below 0.05 for at least one pathogen are listed. *P*-values above 0.05 and their associated ratios are not shown for clarity. *, **, and *** refer to *p*-values below 0.05, 0.01, and 0.001, respectively (Wilcoxon-Mann-Whitney test). HCN, hydrogen cyanide; PG, peptidoglycan; Rhs, DUF4150, and Tox-GHH2, Pfam domains harbored by some of the Type VI effectors.

Other genes or BGCs were negatively associated with inhibition abilities against some of or all the bacterial pathogens. They are especially involved in the production of T6SS belonging to two groups and of antibacterial proteins, including some S-type pyocins and T6SS effectors.

## 4 Discussion

This study aimed at isolating and characterizing *Pseudomonas* strains displaying inhibitory activity against three lettuce bacterial pathogens, namely *X*. *hortorum* pv. *vitians*, *P*. *cichorii*, and *P*. *carotovorum*. More than 1,200 *Pseudomonas* strains were screened for their inhibition potential against these pathogens using a newly developed inhibition assay, resulting in the identification of 35 *Pseudomonas* strains able to inhibit the growth of some or all three pathogens. Their genomes were entirely sequenced and analyzed in search of a species-level identification and of genes or BGCs potentially responsible for biocontrol, plant-growth promotion, and rhizocompetence traits.

The isolated strains belonging to the *P*. *putida* phylogenomic group displayed on average better *in vitro* inhibitory abilities against the three lettuce bacterial pathogens than those belonging to the *P*. *fluorescens* group (Figure 4). The latter group has been extensively studied for its numerous PGPR isolates, contrary to the former (Höfte, 2021; Costa-Gutierrez et al., 2022). Among the 15 isolated strains belonging to the *P*. *putida* group, various strains carry biocontrol-related genes or BGCs that are almost absent from the isolated strains belonging to the *P*. *fluorescens* group (Figure 3). These genes or BGCs are especially linked to the biosynthesis of 7-hydroxytropolone, putisolvins, xantholysin-like compounds, pyochelin, pseudomonine-like compounds, and unknown molecules produced by non-ribosomal peptide synthases-polyketide synthases (NRPS-PKS) and PKS. These compounds may contribute to some extent to the better inhibitory activity observed in strains belonging to the *P*. *putida* group, compared to those in the *P*. *fluorescens* group. The compound 7-hydroxytropolone is an iron-chelating and antimicrobial molecule whom BGC has been uncovered in the genome of three *Pseudomonas* species so far: *P*. *donghuensis*, “*P*. *qingdaonensis*,” and *P*. *wadenswilerensis* (Jiang et al., 2016; Krzyżanowska et al., 2021). In this study, the associated BGC was found in strains belonging to the first two species and not in the only strain of *P*. *wadenswilerensis* recovered. This compound is involved in the inhibition of the nematode *Caenorhabditis elegans* (Gui et al., 2020), phytopathogenic fungi (Muzio et al., 2020; Tao et al., 2020), and various bacterial phytopathogens, including *Pectobacterium brasiliense* (formerly known as *Pectobacterium carotovorum* subsp. *brasiliense*) (Krzyżanowska et al., 2016; Muzio et al., 2020; Matuszewska et al., 2021). Putisolvins and xantholysins are two cyclic lipopeptides mainly, if not solely, described in *P*. *putida* strains (Kuiper et al., 2004; Li et al., 2013; Höfte, 2021). Putisolvins are involved in swarming of the producing strains and can inhibit biofilm formation *in vitro* in other *Pseudomonas* strains (Kuiper et al., 2004). Their role in the biocontrol of plant pathogens *in planta* remains to be demonstrated (Kruijt et al., 2009). Xantholysins, mainly represented by xantholysin A, are involved in the inhibition of aphids (Lim et al., 2017), as well as of fungal and bacterial pathogens, including *X*. *hortorum* (Li et al., 2013). Five of the six strains carrying the pyochelin BGC belong to the *P*. *putida* group and these five strains display medium to high inhibition levels against the three pathogens studied. Pyochelin is a siderophore that has been extensively studied in the opportunistic pathogen *P*. *aeruginosa* PAO1 (Gross and Loper, 2009), but rarely in *Pseudomonas* strains with biocontrol activity. Its diastereoisomers, enantio-pyochelin, has been more often identified in such strains (Youard et al., 2007; Garrido-Sanz et al., 2016). Pyochelin from the strain PAO1 has been shown to inhibit *in vitro* some plant pathogens belonging to the *Xanthomonas* genus (Adler et al., 2012). In the isolated *Pseudomonas* strains, this points to a potential role of this siderophore in the inhibition of the three lettuce bacterial pathogens. Pseudomonine and pseudomonine-like compounds are siderophores identified in various *Pseudomonas* strains of biocontrol interest (Mercado-Blanco et al., 2001; Loper et al., 2012; Biessy et al., 2019; Oluwabusola et al., 2021). They share structural similarities with pyochelin (Gross and Loper, 2009). Some of these compounds have been shown to inhibit the human bacterial pathogen *Mycobacterium tuberculosis in vitro* (Oluwabusola et al., 2021). Another family of BGCs found in the genome of the 35 strains under study pertains to NRPS and PKS. NRPS are large multimodular megaenzymes known to produce a great diversity of bioactive secondary metabolites in bacteria and fungi, including cyclic lipopeptides (Hur et al., 2012). PKS produce various bioactive compounds as well, such as 2,4-diacetylphloroglucinol (DAPG), and encompass, but are not limited to, large and modular enzymes (Gross and Loper, 2009). NRPS and PKS act sometimes in combination to synthesize compounds, such as the antibiotic pyoluteorin. These BGCs may be involved in the biosynthesis of undescribed compounds displaying biocontrol activity. Analytical chemistry and mutagenesis approaches are needed to decipher their products and potential role(s) for their producing strains.

*In vitro* inhibition of the three pathogens *X*. *hortorum* pv. *vitians*, *P*. *cichorii*, and *P*. *carotovorum* by some of the isolated *Pseudomonas* strains positively correlates with the presence of genes involved in pyochelin and T6SSs biosynthesis (Table 2). These might be involved in pathogen inhibition. Pyochelin has already been discussed in this paper because of the presence of its BGC in strains almost exclusively belonging to the *P*. *putida* group. As for the T6SSs, they are membrane-embedded contractile nanomachines that secrete often toxic effectors acting in the periplasm or cytoplasm of target cells (Bernal et al., 2018; Jurënas and Journet, 2021). A T6SS has especially been shown to mediate leaf protection by *P*. *putida* KT2440 against *X*. *campestris* in *Nicotiana benthamiana* (Bernal et al., 2017). However, T6SS-mediated inhibition is often considered as a contact-dependent mechanism (Coulthurst, 2019). In our inhibition assays, we focused on the diameter of the inhibition zone, which would not uncover any potential contact-dependent inhibitory effect usually expected with T6SSs. This would explain why the presence of the T6SS effectors searched for in the genomes of the 35 *Pseudomonas* strains did not positively correlate with their inhibition abilities against the three pathogens. It would potentially point to another role of the T6SSs whose presence in the genomes positively correlates with inhibition abilities. In some cases, T6SSs have been shown to mediate a remote effect, especially through iron acquisition. Chen et al. (2016) have shown that a *Pseudomonas* strain isolated from soil, *P*. *taiwanensis* CMS^T^, was able to use its T6SS to secrete newly synthesized pyoverdine, a siderophore, from the periplasm to the extracellular medium. This T6SS-mediated secretion of pyoverdine contributed to the antagonistic activity of this strain against *X. oryzae*, a rice pathogen. Interestingly, almost all 35 strains carry the BGC responsible for pyoverdine production. Besides, Lin et al. (2017) have shown in *P*. *aeruginosa* PAO1 that the T6SS could also act with a Fe(III)-pyochelin receptor to retrieve iron. This strain uses a T6SS to secrete a protein, TseF, that is incorporated into outer membrane vesicles, where it interacts with the high iron-affinity compound called the *Pseudomonas* quinolone signal. The protein also interacts with the Fe(III)-pyochelin receptor FptA and the porin OprF, allowing the strain to transfer iron into its cytosol. The presence of the pyochelin BGC correlates with *in vitro* inhibition of the three plant pathogens as well, suggesting that this T6SS-mediated mechanism might be used by some strains to inhibit the pathogens. Removing T6SS- and/or pyochelin-related genes in some strains while cultivating the resulting mutants in different iron concentrations would determine whether this iron-dependent inhibition is at play.

The presence of genes involved in HCN and tailocins production is linked to antagonistic activity against *P*. *carotovorum* only. HCN is a toxic volatile compound affecting a wide array of organisms by disrupting the functions of the cytochrome c oxidase, involved in the respiratory chain, and of other metalloproteins (Gross and Loper, 2009). The biosynthesis of this antimicrobial molecule has been demonstrated in numerous *Pseudomonas* strains (Ramette et al., 2003; Flury et al., 2017), and its conserved BGC was found in the genome of numerous strains belonging to this genus (Loper et al., 2012; Garrido-Sanz et al., 2016; Biessy et al., 2019). The compound is actively involved in the inhibitory activity of several *Pseudomonas* strains against a broad range of plant parasites/pathogens, such as insects, nematodes, fungi, and bacteria (Höfte, 2021). Such activity has been seldom reported against bacterial phytopathogens. In 2012, Lanteigne et al. (2012) demonstrated that the strain *P*. *brassicacearum* LBUM300 inhibited the bacterial plant pathogen *Clavibacter michiganensis* subsp. *michiganensis* through the production of HCN and other antibiotics. However, another research team found no correlation between HCN production and the inhibition of bacterial plant pathogens by some rhizobacteria, including *P*. *carotovorum* and *X. campestris* pv. *campestris* (Rijavec and Lapanje, 2016). Tailocins are phage tail-like antibacterial proteins found in various genera belonging to the *Pseudomonadota* phylum, including the genus *Pseudomonas* (Ghequire and De Mot, 2014; Príncipe et al., 2018). They directly induce a depolarization of the membrane of their targets, leading to cell death. These proteins are known to affect closely related bacteria, usually belonging to the same genus or species (Scholl, 2017), but have sometimes been demonstrated to inhibit bacteria outside of their genus, such as the plant pathogen *Erwinia amylovora* (Jabrane et al., 2002; Ghequire and De Mot, 2014). To our knowledge, *P*. *carotovorum* has never been shown to be affected by tailocins. However, two bacteriophages, PP1 and PP16, effectively inhibit this pathogen and represent promising biocontrol tools (Lim et al., 2013; Voronina et al., 2019). Given the structural similarity between tailocins and bacteriophages, tailocins produced by some *Pseudomonas* strains might be affecting *P*. *carotovorum*. The potential role of tailocins and HCN in pathogen inhibition should be assessed through a reverse genetics approach.

The 35 inhibitory *Pseudomonas* strains display a high degree of diversity, encompassing two phylogenomic groups, six subgroups, and at least 27 species. Many of these species are represented by few if any strains of biocontrol interest, such as *P*. *laurylsulfatiphila* and *P*. *sichuanensis*, and have been recently described (Furmanczyk et al., 2018; Qin et al., 2019; Girard et al., 2021). Some other strains isolated in this study belong to species well known for their numerous PGPR representatives, such as *P*. *brassicacearum*, *P*. *protegens*, and *P*. *putida* (Paulin et al., 2017; Nelkner et al., 2019; Höfte, 2021; Costa-Gutierrez et al., 2022). This diversity arose thanks to the numerous soil sample collection sites and the discriminating strain isolation process, based only on a *Pseudomonas*-selective medium and a binary inhibition criterion against the three lettuce bacterial pathogens. Such diversity has led to the identification of a wide range of plant growth promotion-related genes and BGCs in the genomes, heterogeneously distributed among the strains. This high heterogeneity made difficult the identification of correlations between the presence of some genes or BGCs and the inhibitory abilities of the *Pseudomonas* strains, but potentially expanded the number of biocontrol mechanisms at work against the plant pathogens studied. Furthermore, the presence of a BGC in a genome does not necessarily indicate whether and how much of the corresponding compound is produced (Biessy et al., 2021). Better understanding these potentially diverse mechanisms and biosynthesis pathways may be central to develop effective *Pseudomonas*-based biocontrol strategies against bacterial pathogens in lettuce fields.

## 5 Conclusion

A novel assay was developed to enable the rapid screening of 1,210 *Pseudomonas* strains for their antagonistic properties against three lettuce bacterial pathogens. Among these, 35 *Pseudomonas* strains identified for their *in vitro* inhibitory abilities against these pathogens are promising. Many biocontrol-related genes and BGCs have been identified and are potentially involved in various mechanisms to inhibit the targeted pathogens. These mechanisms will have to be validated by using reverse genetics and analytical chemistry approaches, while performing greenhouse and field experiments to determine the most effective strains to prevent bacterial disease development in an agricultural context. This work paves the way to sustainable and preventive lettuce protection methods against bacterial pathogens and contributes to the expansion of quality genomic data for *Pseudomonas* species, especially those for which little information is available.

## Data availability statement

The complete genome sequences of the 35 *Pseudomonas* strains have been deposited at DDBJ/ENA/GenBank under the accession numbers provided in Table 1. PacBio sequencing reads have been deposited into the Sequence Read Archive (BioProject PRJNA779451) under the accession numbers provided in Supplementary Table 2. The genome versions described in this manuscript are the first versions.

## Author contributions

AZ, AB, MCi, MCa, DA, and MF: conceptualization. AB, MCi, MCa, and MF: methodology. JB: software. AZ, AB, MCi, and MCa: formal analysis. AZ, AB, MCi, MCa, and DA: investigation. AB: data curation. AZ and AB: writing—original draft and visualization. AZ, AB, MCi, MCa, DA, JB, and MF: writing—review and editing. MF: supervision and funding acquisition. All authors contributed to the article and approved the submitted version.
