# Supplementary figures and images for: Harnessing the genomic diversity of Pseudomonas strains against lettuce bacterial pathogens
Source: Front Microbiol. 2022 Dec 22;13:1038888. doi: 10.3389/fmicb.2022.1038888 (PMC9814014; doi:10.3389/fmicb.2022.1038888)

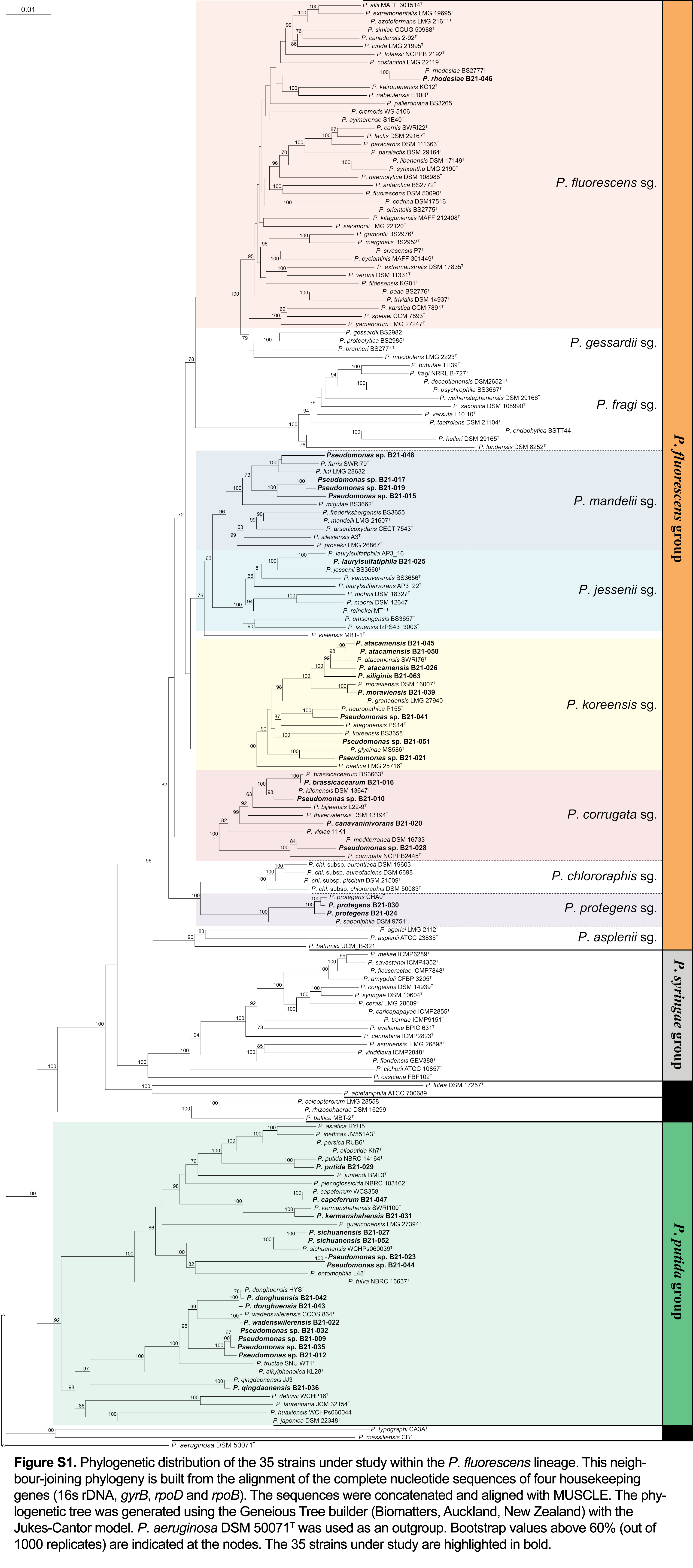

Supplement: Supplementary file 6 [file Image_1.tif]

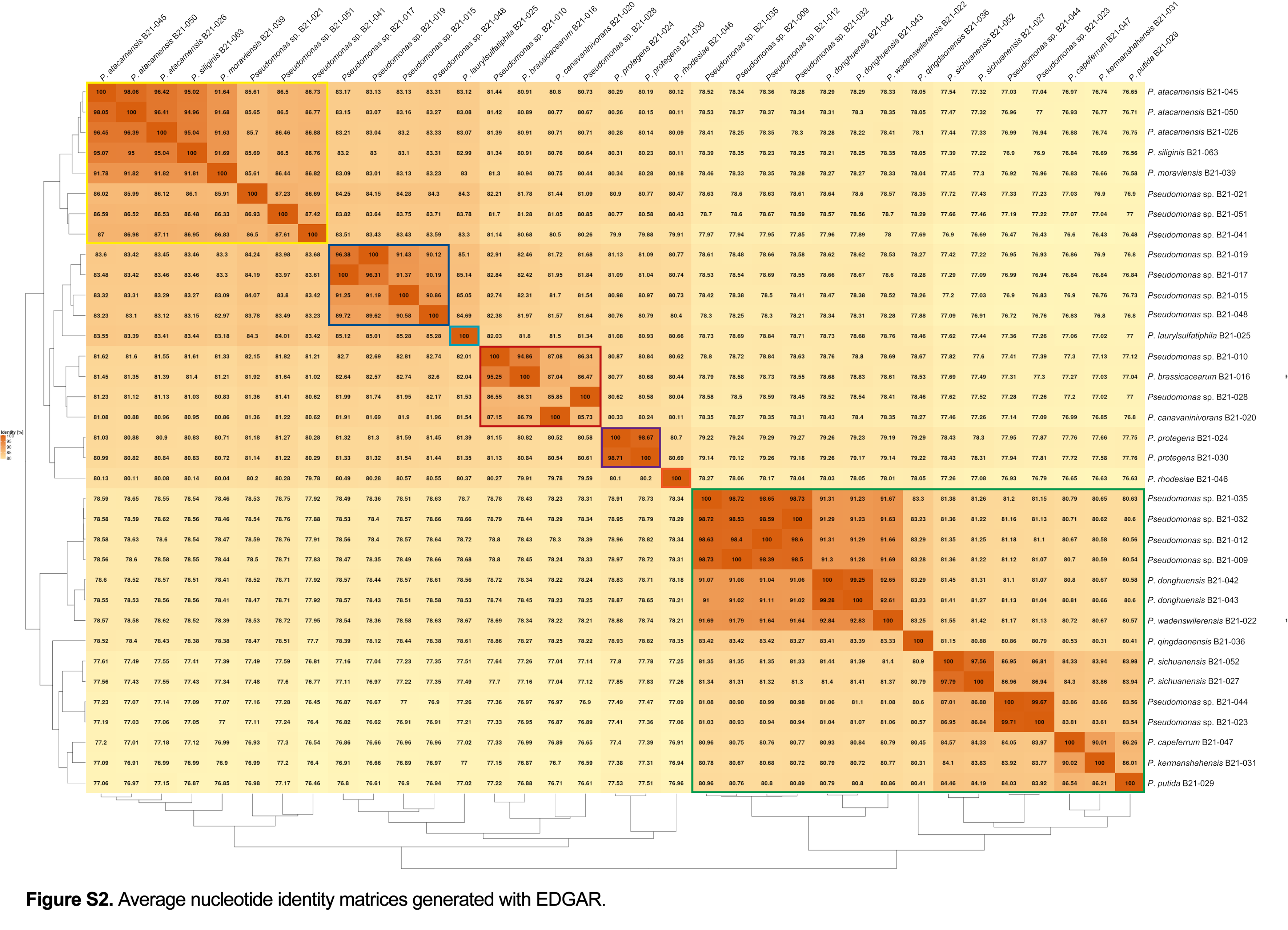

Supplement: Supplementary file 7 [file Image_2.tif]

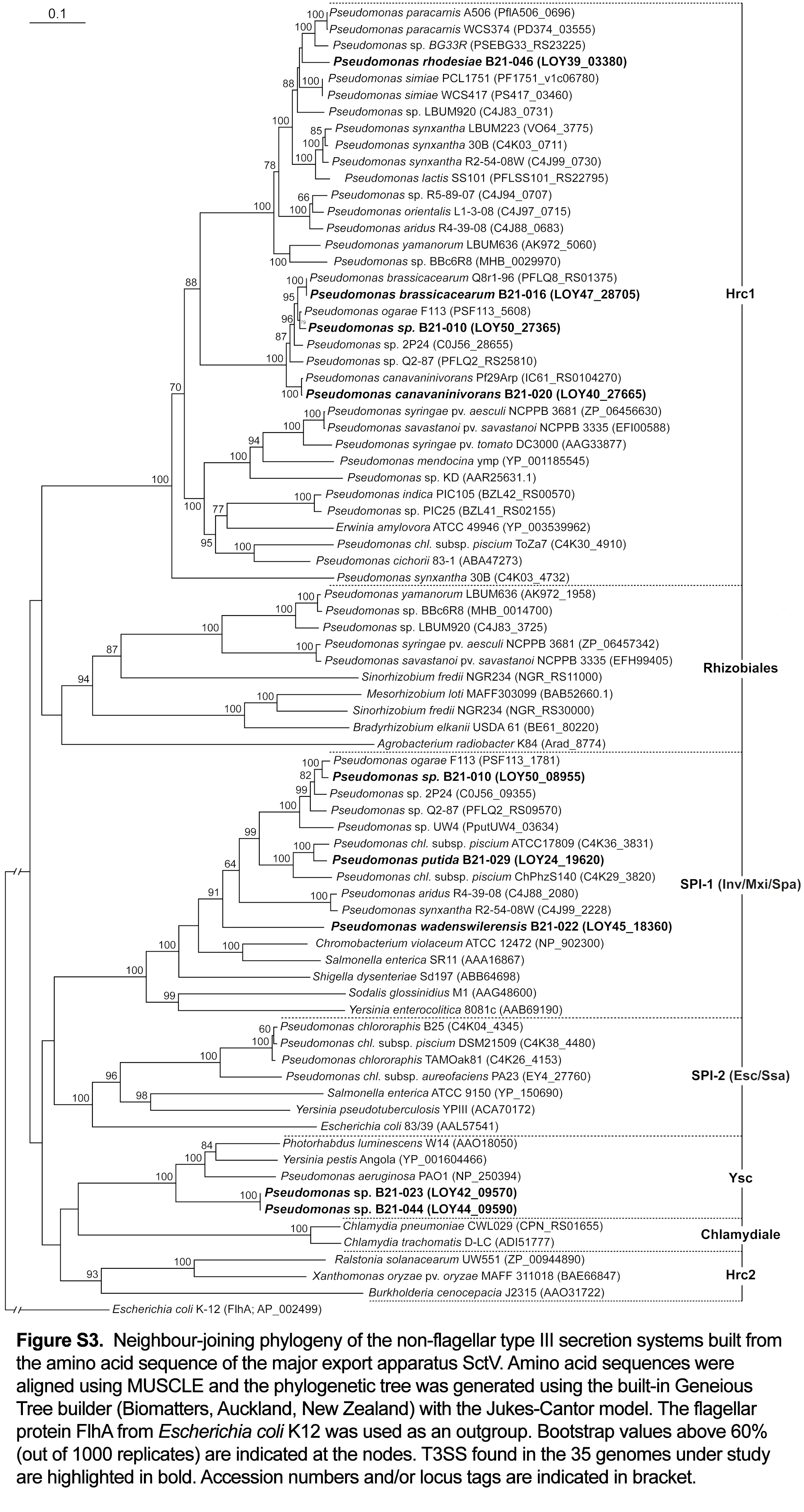

Supplement: Supplementary file 8 [file Image_3.tif]

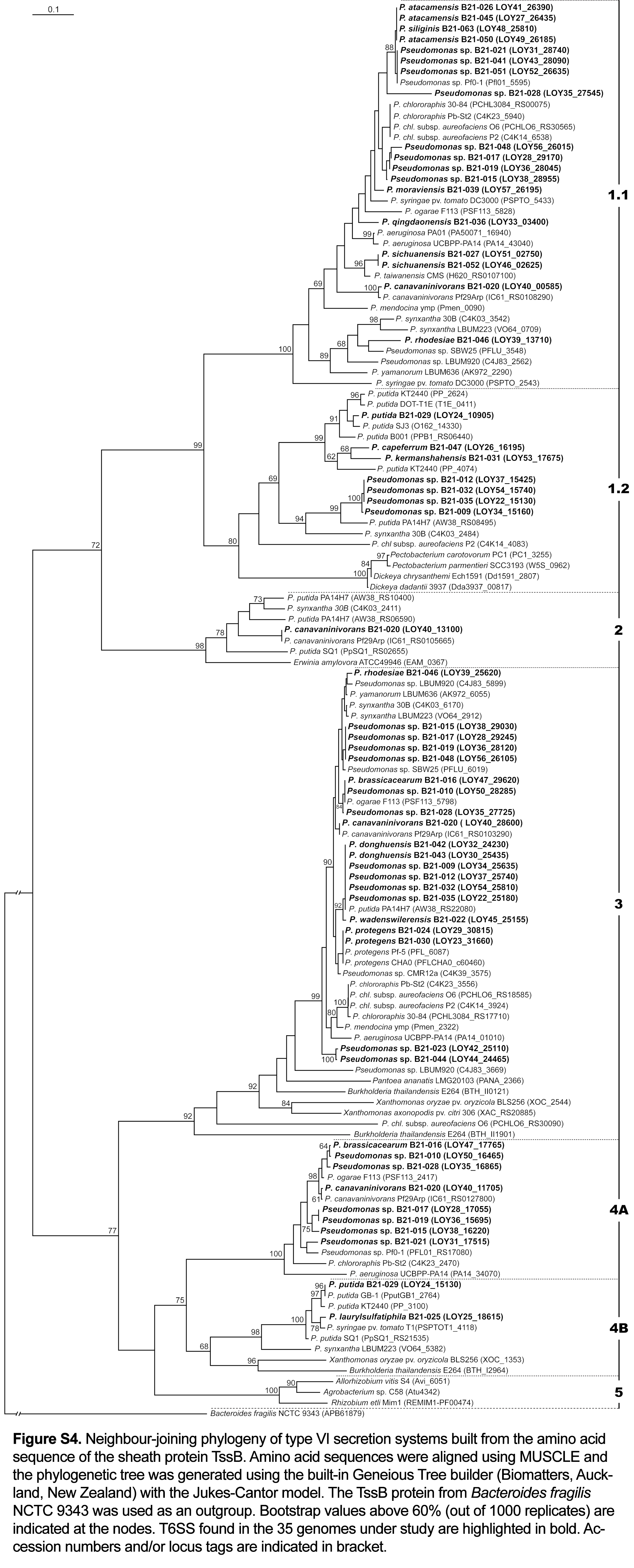

Supplement: Supplementary file 9 [file Image_4.tif]
